# Survey and Molecular Diagnostics of Target Site Mutations Conferring Resistance to Insecticides in Populations of *Aphis spiraecola* from Greece

**DOI:** 10.3390/insects16121199

**Published:** 2025-11-25

**Authors:** Aris Ilias, Panagiotis J. Skouras, Argyro Kalaitzaki, Emmanouil Roditakis, Evangelos Tsirikos, Anastasia Tsagkarakou, John Vontas, John T. Margaritopoulos

**Affiliations:** 1 Institute of Olive Tree, Subtropical Crops and Viticulture, Hellenic Agricultural Organization “DEMETER”, 41335 Heraklion, Greece; kalaitzaki@elgo.gr (A.K.);; 2 Department of Agriculture, School of Agricultural Sciences, Hellenic Mediterranean University, 72100 Heraklion, Greece; eroditakis@hmu.gr; 3 Laboratory of Agricultural Entomology and Zoology, Department of Agriculture, University of the Peloponnese, 24150 Kalamata, Greece; p.skouras@uop.gr; 4 Agrotopos Agricultural Products, 21100 Nafplion, Greece; v.tsirikos@gmail.com; 5 Institute of Molecular Biology and Biotechnology, Foundation for Research and Technology Hellas, 70013 Heraklion, Greece; 6 Department of Crop Science, Agricultural University of Athens, 11855 Athens, Greece; 7 Institute of Industrial and Fodder Crops, Hellenic Agricultural Organization “DEMETER”, 11145 Volos, Greece

**Keywords:** spirea aphid, green citrus aphid, insecticide resistance, molecular diagnostics

## Abstract

The spirea aphid, *Aphis spiraecola* (Hemiptera: Aphididae), is a major pest of citrus and other crops, causing feeding damage and transmitting plant viruses. Control relies mainly on insecticides, increasing the risk of resistance. We surveyed Greek populations of *A. spiraecola* (2022–2023) and developed molecular diagnostics to detect target site mutations linked to resistance against neonicotinoids, carbamates, organophosphates, pyrethroids, and spirotetramat. Seventy-two aphids were analyzed. All individuals were homozygous and susceptible at R81T, A302S, L1014F, M918 variants, and A2226V loci. However, the S431F mutation in the *AChE2* gene, associated with resistance to dimethyl-carbamates, such as the selective aphicide pirimicarb, was present in all regions. Heterozygotes occurred at high frequencies, while homozygous resistant individuals were rare. These findings indicate that Greek populations remain largely susceptible to most insecticide classes, but the widespread occurrence of S431F questions the use of pirimicarb in resistance management. The diagnostic assays developed here are cost-effective tools for large-scale monitoring, enabling early detection of resistance in *A. spiraecola*. Continued surveillance, combined with bioassays and studies on metabolic mechanisms, will be essential for sustainable pest management.

## Simple Summary

The spirea aphid, *Aphis spiraecola*, is a small insect that damages citrus trees and other crops by feeding on plant sap and spreading viruses. Farmers typically rely on insecticides to manage this pest; however, excessive use can result in the development of resistance, thereby reducing the effectiveness of control measures. In this study, we collected aphids from citrus orchards across Greece and tested them for genetic changes already known to confer insecticide resistance in related species. We found that Greek populations do not yet carry mutations conferring resistance to most common insecticides, such as neonicotinoids, pyrethroids, organophosphates, and spirotetramat. However, one mutation (S431F), which can reduce the effectiveness of dimethy-carbamate insecticides, such as pirimicarb, was detected at high frequency. This suggests that pirimicarb, a selective aphidicide, although not registered for citrus in Greece, may no longer be a reliable option in cases of emergency use or in resistance management programs. We also developed simple and cost-effective diagnostic tools that can quickly identify resistance mutations in field samples. These tools may enable farmers and advisors to monitor aphid populations more effectively and support sustainable pest control strategies in citrus production.

## 1. Introduction

Aphids (Hemiptera: Aphididae) are phytophagous insects, with more than 5000 species described so far, but only about 100 species pose a serious risk to many crops, causing major yield losses and their reputation as key pests in agriculture [1]. These phloem-feeding insects impact plants both directly and indirectly. They directly harm plants by sucking essential nutrients from the phloem, which can result in wilting and lower yields. Indirect damage comes from their honeydew extracts, associated with the growth of fungal pathogens, and the spread of viral diseases. One notable species is the green citrus aphid or spirea aphid, *Aphis spiraecola* Patch (Hemiptera: Aphididae). This small, greenish-yellow aphid is quite polyphagous, with hosts from more than 20 plant families. The species shows a worldwide distribution, and its most important crop hosts are probably apples and *Citrus.* Apart from the direct damage that this species causes, it also transmits some plant viruses, including *Citrus tristeza virus* (CTV) [1]. Although not a particularly efficient vector of CTV, the aphid presumably contributes significantly to the spread of the virus, in regions where it develops into large populations (e.g., Greece, Morocco) [2,3].

The management of this pest largely relies on chemical insecticides, but this approach may lead to the development of resistant aphid populations. This scenario is quite common in several aphid pests, with two notable examples being the peach-potato aphid, *Myzus persicae* (Sulzer) and the cotton melon aphid, *Aphis gossypii* Glover (both Hemiptera: Aphididae). In these species, the mechanisms responsible for resistance to insecticides have been well studied [4,5,6].

Similarly to other insects, the resistance of aphids to insecticides, as stated in the literature, is largely dominated by two primary mechanisms, i.e., target site resistance and metabolic resistance [6]. Target site mutations are associated with amino acid substitutions in the proteins, which is the target of insecticides. In *M. persicae* and *A. gossypii*, the substitution of arginine to threonine (R81T) in the *β*1 subunit of the nicotinic acetylcholine receptor is considered a key mechanism for neonicotinoid resistance [6,7,8]. Mutations, along with differential expression of acetylcholinesterase genes (*ACE1* and *ACE2*) between susceptible and resistant aphids, confer insensitivity to carbamate and organophosphate insecticides [6,9,10,11]. In addition, the knockdown resistance (*kdr*) mutation (L1014F) and the super-knockdown resistance (*skdr*) mutations (M918T/L/V/Ι) in the voltage-gated sodium channel (*VGSC*) are associated with resistance to pyrethroid insecticides [6,12,13,14,15]. Finally, resistance to spirotetramat has been linked to a single non-synonymous mutation that causes an alanine-to-valine substitution (A2226V) in a highly conserved region of the *ACC* carboxyltransferase domain [16].

In the literature, few studies report the development of insecticide resistance in *A. spiraecola* compared to other aphid species. George et al. [17], using leaf dip bioassays and insecticides from different chemical groups, examined field-collected *A. spiraecola* samples from India and found zero or very low Resistance Factors (RFs). Despite this, some historical accounts document that *A. spiraecola* exhibits tolerance or resistance to older insecticide classes. Smirle et al. [18] reported that esterase activity in *A. spiraecola* clones from British Columbia and Washington State was positively correlated with their response to pirimicarb and dimethoate. Similarly, Powell et al. [19] found that in six-year field experiments in a Florida citrus grove, imidacloprid treatments controlled the aphid populations, while treatments with aldicarb or oxydemeton-methyl did not.

Recently, widespread resistance to pyrethroids lambda-cyhalothrin, bifenthrin, permethrin, fenvalerate, and deltamethrin has been documented in *A. spiraecola* populations in major apple-growing regions of China, with Resistance Factors (RFs) up to 384, 94, 349, 565, and 315-fold, respectively [20,21]. The authors reported that this resistance was associated with P450 activity and *kdr* (L1014F) and *skdr* (M918V/L) mutations. In another study on Chinese populations, Tang et al. [22] reported one population with an RF of 42 to abamectin and another with an RF of 69 to thiamethoxam. Most populations showed low to moderate resistance to the three neonicotinoids examined (imidacloprid, acetamiprid, thiamethoxam). The authors did not find any of the known resistance mutations in the target receptors (*nAChR*, *GABA*, or *GluCl*), and they suggested that other mechanisms, such as metabolic detoxification, are likely responsible.

In Greece, *A. spiraecola* is a major pest of citrus orchards and is considered one of the main aphid species contributing to the CTV spread [2]. As far as we know, there are no published studies on the insecticide resistance status of *A. spiraecola* populations from Greece, and recent data are lacking. Furthermore, the presence and distribution of resistance-linked mutations in Greece remain undocumented. Recognizing the importance of monitoring and early detection of insecticide resistance mutations for the effective management of the pest, we developed rapid and cost-effective molecular diagnostic tools and analyzed several samples of *A. spiraecola* from Greece, collected in 2022 and 2023.

## 2. Materials and Methods

### 2.1. Aphid Material

**Figure 1:**
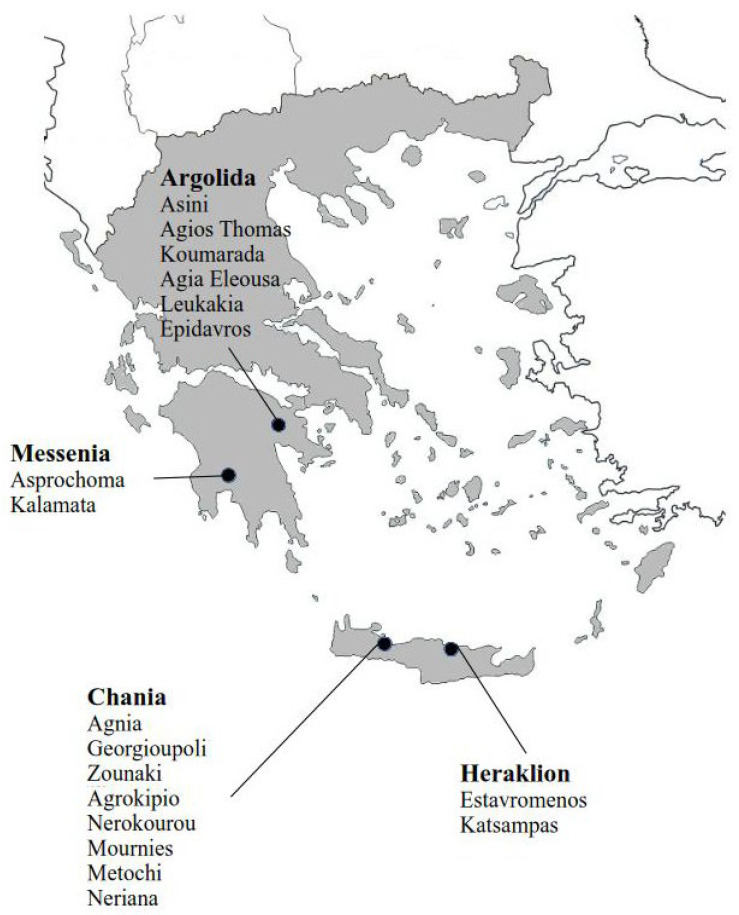
Collection sites of *Aphis spiraecola* samples in prefectures of Greece: Argolida (*N* = 11), Messinia (*N* = 7), Heraklion (*N* = 12), and Chania (*N* = 42).

Seventy-two *A. spiraecola* samples were collected from citrus trees from various locations in Greece during the years 2022–2023. Five samples were from domestic gardens and 67 from citrus cultivations. The origin of the samples, the collection date, and the host plants are summarized in Table 1, while the location of the samples is shown in Figure 1. Aphids were randomly collected from each infested tree, preserved in absolute ethanol and stored at −20 °C until use.

### 2.2. Genomic Extraction, PCR Amplification of Gene Targets and DNA Barcoding

Genomic DNA was extracted from individual adult female aphids, using DNAzol reagent (Molecular Research Center, Inc., Cincinnati, OH, USA) according to the manufacturer’s instructions. Briefly, an individual aphid was placed in 200 μL reagent in an Eppendorf tube and was homogenized using a plastic-pestle homogenizer. The tubes were centrifuged at 10,000 rpm for 10 min at room temperature (RT), the supernatant was transferred into a new 1.5 mL tube, and the DNA was precipitated with an equal volume of 100% ethanol. After undergoing centrifugation at 13,000 rpm for 20 min at RT, the supernatant was discarded, and the DNA pellet was washed with 1 mL of 75% ethanol. Lastly, the tubes were centrifuged at 13,000 rpm for 5 min at RT, the supernatant was discarded, and the DNA pellet was re-suspended in 30 μL sterile water.

Genomic DNA was used as template for PCR amplification of gene fragment of COI (DNA barcoding) and gene fragments encompassing insecticide resistance mutations using the specific primers listed in Table 2. PCR reaction (25 μL) contained 2 μL genomic DNA, 0.4 μM primers, 0.2 mM dNTPs, 2.5 μL of 10× buffer, and 1U Taq DNA polymerase (Enzyquest, Heraklion, Greece). The thermal conditions were as follows: 94 °C for 2 min followed by 35 cycles of 94 °C for 30 s, 52–55 °C for 30 s, 72 °C for 45 s, with a final extension step for 2 min at 72 °C. PCR products were purified using NucleoSpin Extract II (Macherey-Nagel, Düren, Germany) and sequenced directly on PCR product with the original PCR primers (Table 2).

Sequencing reactions were performed at the GENEWIZ sequencing facility (GENEWIZ Germany GmbH, Bahnhofstrasse 86, 04158 Leipzig, Germany). Sequences were analyzed with Bioedit [23]. The presence/absence of target site mutations was based on visual examination of sequence chromatographs.

Species identification (COI) was performed by subjecting all 72 individuals (1 aphid per field sample) used in this study. The same individuals were analyzed for the presence of insecticide resistance mutation. It should be noted that to ensure the reliable design of the molecular diagnostics, we investigated the presence of polymorphisms in all the gene targets from 20 individuals from different localities (randomly selected) via sequencing.

### 2.3. Development of RFLP Diagnostics for the Detection of Susceptible A. spiraecola Alleles in Resistance Gene Targets

RLFP diagnostic assays were designed to detect the susceptible (wt) alleles of A302S, R81T, L1014F, and A2226V resistance-associated positions (*AChE*, *nAChR β*1, *vgsc* and *ACCase* genes, respectively).

The primers Ag_AChE2_F1 and Ag_AChE2_R1 (Table 2) were used for the detection of the A302 wt allele. An amplicon of 897 bp of *AChE2* was produced, using the aforementioned PCR conditions, encompassing both A302S and F431W resistant positions. The Cac8I (GCNNGC) restriction enzyme, recognizes one site in A302 wt allele, yielding in a restriction pattern of two fragments (584 and 313 bp) (Figures S1 and S5a).

Primers Ag_nAChR_β1_F1 and Ag_nAChR_β1_R1 (Table 2) were used for the detection of the R81 wt allele. An amplicon of 508 bp of *nAChR_β1* containing R81T resistant position was produced, with the PCR conditions described previously. The BsmAI (GTCTC) restriction enzyme recognizes one site in the R81 wt allele, yielding in a restriction pattern of two fragments (318 and 190 bp) (Figures S2 and S5a).

For *vgsc*, all individuals were genotyped by sequencing, since we were not able to develop a simple PCR-RFLP diagnostic assay to discriminate for the M918T/L/V/I susceptible allele. To detect the L1014F wt allele, a PCR-RFLP diagnostic assay was designed using the primers Ag_vgsc_F1 and Ag_vgsc_R1 (Table 2). An amplicon of 627 bp of *vgsc* containing both L1014F and M918T/L/V/I resistant positions was produced with the PCR conditions described previously. The BstEII (GGTNACC) restriction enzyme, recognizes three sites in L1014 wt allele, yielding in a restriction pattern of four fragments (325, 205, 91 and 6 bp) (Figures S3 and S5a). Primers Mp_Ag_ACCase_F1 and Mp_Ag_ACCase_diaR (Table 2) were used for the detection of the A2226 wt allele. An amplicon of 126 bp of *ACCase* was produced, with PCR conditions described previously, containing A2226V resistant position. The reverse primer creates a restriction site for the MwoI enzyme and, when combined with the forward primer, enables the detection of the susceptible A2226 allele. The MwoI (GCNNNNNNNGC) restriction enzyme, recognizes one site in the modified susceptible (wt) A2226 allele, yielding in a restriction pattern of two fragments (102 and 24 bp) (Figures S4 and S5b).

**Figure 2:**
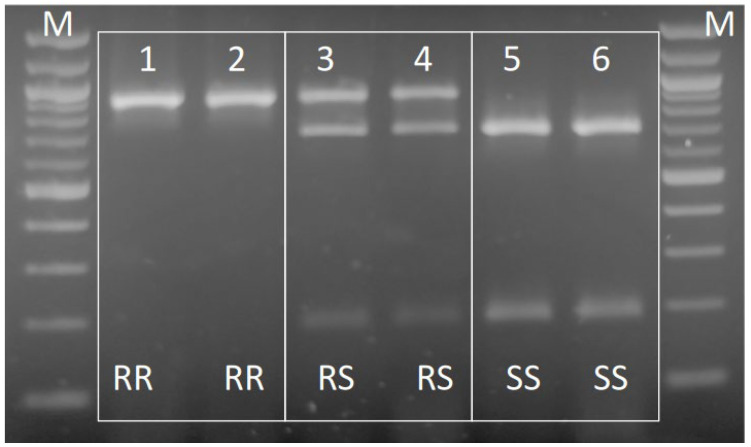
PCR-RFLP diagnostic assay of the S431F resistance mutation in *A. spiraecola* after SspI enzymatic digestion. Lanes 1, 2: homozygousr individuals (RR); lanes 3, 4: heterozygous individuals (SR); lanes 5, 6: homozygous susceptible individuals (SS). M: molecular weight marker (Quick-Load 100 bp DNA Ladder, New English Biolabs, Frankfurt, Germany).

In the case of S431F mutation, an RLFP diagnostic assay was designed, and distinguished the resistant (F431) and the susceptible (S431) alleles. The assay is based on the PCR amplification of an 879 bp fragment of *AChE2* gene encompassing S431F and A302S mutation sites using primers Ag_AChE2_F and Ag_AChE2_R (Table 2), with conditions described previously, and subsequent restriction digest with SspI. The 431F disrupts the restriction site for SspI enzyme (AATATT), which is present in the S431 wt allele (Figure 2 and Figure S1). Therefore, SspI yields a restriction pattern of one undigested fragment (897 bp) for the resistant allele F431 and two fragments (702 and 195 bp) for the susceptible S431 allele, enabling the reliable genotyping scoring.

Digestion protocols in all cases were performed using NEB restriction enzymes (NEW ENGLAND BIOLABS, Frankfurt, Germany) according to manufacturers’ instructions, and products in all cases were visualized in 2% (*w/v*) agarose gel, apart from *ACCase* which was electrophoresed in 3.5% *w/v*.

### 2.4. Statistical Analysis

Deviation from Hardy–Weinberg equilibrium (HWE) at the S431F locus was examined using the Probability-test as implemented in GENEPOP version 4.7.5, accessed on 30 October 2025 (http://genepop.curtin.edu.au) [24].

## 3. Results and Discussion

One key component of Insecticide Resistance Management strategies, in the framework of IPM, is the early detection of insecticide resistance mechanisms in the pest populations [6,25,26,27]. Furthermore, insecticide resistance is a dynamic phenomenon in space and time, with many notable examples among aphid species [8,28,29,30,31], and therefore, continuous monitoring is necessary.

### 3.1. New Molecular Diagnostics for Target Site Resistance Mechanisms

To address the aforementioned challenges, we developed and applied reliable, cost-effective molecular diagnostics that do not require complex equipment. Before applying molecular diagnostics Cytochrome oxidase I (*COI*)–based species identification was performed for all 72 individuals (one aphid per field sample) included in this study. All specimens were identified as *A. spiraecola*, exhibiting 100% sequence similarity to reference sequences available in public databases (BOLD and GenBank). Regarding gene targets, the sequences analyzed in the present study showed a high nucleotide conservation in individual aphids from all sampling localities. No polymorphisms were observed in the genes of interest, except for the F431S resistance mutation, across all sequenced individuals. Due to the lack of documented resistance mutations in the sequences analyzed (R81T, A2226V, and S302, L1014F), we designed RFLP-based diagnostic tools to detect the wild-type alleles corresponding to the four resistance mutations. Given the high nucleotide conservation of *vgsc*, *AChE*, *nAChR β1*, and *ACCase* genes within and between aphid species, we anticipate that these diagnostic tools will enable the future detection of any emergent resistance alleles. Nevertheless, prior to applying these diagnostics in other countries, it is advisable to verify sequence conservation through sequencing. It is worth noting that our design successfully distinguished the recently identified F1014L mutation reported in China [20,21], which has not yet been detected in Greece. The resistance-associated 1014F allele produced a fragment profile of 416–205–6 bp, whereas the wild-type allele yielded 325–205–91–6 bp. To our knowledge, this study is the first to simultaneously examine the most critical aphid resistance mutations in the spirea aphid. Furthermore, the sequences of the target genes, which were previously unavailable in public databases, provide a valuable resource for future studies on this species.

### 3.2. Frequency of Insecticide Resistance Mutations

All the examined aphid individuals were found to be homozygous susceptible (SS) for the R81T, A2226V, M918L/T/VI, L1014F and A302S resistance mutations. However, the S431F mutation was detected in all sampling regions (Table 3). Homozygous resistant genotypes were found only in the Chania region at a low frequency (3%), while heterozygous genotypes were found in all regions. In three of these regions (Heraklion, Chania, Messinia), they were found at high frequencies, ranging from 66.7 to 100%. The frequency of the resistant allele (fR) in these regions ranged from 33.3 to 50.0% (total for all regions 37.5%). For HWE, we only examined the population from Chania, from which an adequate number of aphids were analyzed. This population showed significant deviation from HWE (*p* < 0.001); this is associated with heterozygote excess (F*_IS_* = −0.627). In order to avoid any Wahlund effect (i.e., joining samples from different genetic pools) that might affect F*_IS_* and HWE estimations, we did not test for HWE for the total sample of aphids. In the literature, cases of documented heterozygote excess [25] or medium to high frequencies of homozygous resistant and/or heterozygous individuals have been recorded in other aphid species [8,32,33,34]. One possible explanation for the HWE deviation reported here is that modified *AChE* has been found to be associated with moderate to strong fitness cost in aphids (e.g., lower reproductive rates or competitive fitness) [35,36], as well as in other insect pests [37], and thus homozygous resistant genotypes may be selected against, especially in periods without selection pressure.

The data of these resistance mutation frequencies suggest that the spirea aphid, in the citrus growing regions of Greece, has not yet acquired the genetic background to combat neonicotinoid, pyrethroid, and organophosphate insecticides, as well as spirotetramat. However, the widespread presence of the S431F mutation, which is associated with resistance to dimethyl-carbamates in other aphid species, suggests that the efficacy of pirimicarb might be compromised. Although pirimicarb has not been registered for use in citrus for the past decade, its inclusion in insecticide rotation strategies would be questionable, even in emergency situations.

In the present study, we did not examine the potential metabolic mechanisms known to reduce the efficacy of the aforementioned chemical groups against other aphid species. While such analyses are beyond the scope of this work, we plan to address them in future research, in conjunction with insecticide bioassays not performed in the present study.

In general, *A. spiraecola* is not considered a difficult-to-control insect pest due to the development of insecticide resistance. Foster et al. [4], in their review, did not include *A. spiraecola* among the insecticide-resistant aphid species. To our knowledge, significant cases of resistance and target site mutations have been reported exclusively in Far East populations of *A. spiraecola*. These studies reported resistance to pyrethroids, neonicotinoids, and abamectin [20,21,22]. However, given the invasive abilities of aphids [32] and the documented evolutionary events often related to insecticide resistance, in perceptible timescale [33,34], continuous monitoring of spirea aphid populations is crucial, even in areas where control programs are currently effective. To support this effort, the molecular diagnostics presented in our study could be valuable tools for the rapid and cost-effective mass screening of *A. spiraecola* samples from various hosts and regions and to feed information into the decision systems in the framework of Integrated Pest Management programs.

One question that arises from our data is how spirea aphid populations show high frequencies of the resistant S431F allele in the absence of selection by pirimicarb during the last years. There are two possible theories to explain this “paradox”. First, a recent introduction of resistant genotypes from abroad, which then spread in various regions of Greece. The spirea aphid is considered anholocyclic through most of the world, but holocycles involving host-alternation (*Spiraea* spp. as primary hosts) has been reported from North America, Brazil, and Japan [38,39]. Thus, one might expect the introduction of heterozygous and homozygous resistant genotypes. Surveys are needed in populations from neighboring countries to validate this hypothesis. The second hypothesis, and maybe the most probable, is that the extensive use of pirmicarb during the previous decade had selected resistant genotypes in citrus orchards in Greece, which then proliferated through asexual reproduction and outnumbered the susceptible genotypes. It is possible the relaxation period of selection pressure is not enough for a reversion from resistant to susceptible populations, given the probable involvement of any fitness cost.

## 4. Conclusions

In conclusion, our study provides evidence that *A. spiraecola* in Greece does not yet possess known target site mechanisms for resistance to neonicotinoids, pyrethroids, and organophosphates. We report for the first time in the species the S431F mutation, which is known to be associated with resistance to dimethyl-carbamates. Further research, particularly evolving bioassays, is needed to evaluate the efficacy of the registered insecticides used in Greece for *A. spiraecola* management.

## Figures and Tables

**Table 1:** Details of the *Aphis spiraecola* individuals analyzed in the present study.

Host-Plant	Region			*Total*
	Crete	Argolida	Messenia	
*Citrus japonica*	1			1
*Citrus limon*	18	2		20
*Citrus reticulata*	6	7	4	17
*Citrus sinensis*	29	2	3	34
*Total*	*54*	*11*	*7*	72

**Table 2:** Primers and amplified PCR products used in this study.

Primer Name	Gene	Sequence (5′–3′)	Amplified Product (bp)	Resistance Mutation	Annealing T (°C)
Ag_AChE2_F1	AChE2	TATAAACGTAGTAGTGCCAAGG	897	A302S, S431F	54
Ag_AChE2_R1		CCGACCATTTTGTCCAAAGC			
Ag_nAChR_β1_F1	nAChR_β1	TGCATACGTGGTACGTACATAA	508	R81T	52
Ag_nAChR_β1_R1		TGAACGGTTTGCAGTCAAGC			
Ag_vgsc_F1	VGSC	CTGCGGGTTACCAAGGACTCTC	627	M918L/T/I, L1014F	55
Ag_vgsc_R1		ATCCACCTCGCCGTTTGCAT			
Mp_Ag_ACC_F1	ACCase	AATTTGGTGCATACATTGTTGA	150	A2226V	52
Mp_Ag_ACC_R1		CTGGATCTGCATACATCTCAATA			
Mp_Ag_ACC_diaR		GTCTTGGATTAATAGTAGTAgCTACA	126		
LCO_1490	COI	GGTCAACAAATCATAAAGATATTGG	672	DNA barcoding	52
HCO_2198		TAAACTTCAGGGTGACCAAAAAATCA			

**Table 3:** Frequency (%) of *Aphis spiraecola* genotypes and the resistant allele (fR) for the S431F mutation.

Genotypes	Region				Total
	Argolida	Chania	Heraklion	Messinia	
RR	0.0	4.8	0.0	0.0	2.8
RS	9.1	81.0	66.7	100	69.4
SS	90.9	14.3	33.3	0.0	27.8
*fR*	*4.5*	*45.2*	*33.3*	*50.0*	*37.5*
*N*	*11*	*42*	*12*	*7*	*72*

*N* = number of aphids analyzed.

## Data Availability

All data is provided in the manuscript. The nucleotide sequences generated in this study have been deposited in GenBank under accession numbers PX504462-PX504465.

## Supplementary Materials

The following supporting information can be downloaded at https://www.mdpi.com/article/10.3390/insects16121199/s1. Figure S1. Diagrammatic representation of S431F and A302S PCR-RFP diagnostic assays; Figure S2. Diagrammatic representation of R81T PCR-RFPL diagnostic assay; Figure S3. Diagrammatic representation of L1014F PCR-RFP diagnostic assay; Figure S4. Diagrammatic representation of A2666V PCR-RF diagnostic assay; Figure S5. (a) PCR-RFLP diagnostic assay for the detection of A302, L1014 and R81 wild-type allele; (b) PCR-RFLP diagnostic assay for the detection of A2226 wild-type allele.
