# Supplementary material for: Survey and Molecular Diagnostics of Target Site Mutations Conferring Resistance to Insecticides in Populations of Aphis spiraecola from Greece
Source: Insects. 2025 Nov 25;16(12):1199. doi: 10.3390/insects16121199 (PMC12733833; doi:10.3390/insects16121199)
Supplement: Supplementary file 1 [file insects-16-01199-s001.zip › Fig_S1.pdf]

```
               P   R   P   Q   N   A   A   V   M   V   W   I   F   G   G   G   F   Y   S   G   S   A   T
As_AChE_wt    CCT AGA CCA CAA AAT GCA GCA GTA ATG GTG TGG ATT TTT GGT GGA GGA TTT TAC TCC GGG TCT GCT ACT  69
               L   D   I   Y   D   P   K   I   L   V   S   E   E   N   V   I   L   V   S   M   Q   Y   R
              TTG GAT ATT TAC GAT CCC AAA ATA CTC GTA TCG GAA GAA AAC GTG ATT TTG GTA TCC ATG CAG TAC AGG 138
               V   A   S   L   G   F   L   Y   F   D   T   E   D   V   P   G   N   A   G   L   F   D   Q
              GTC GCG TCA TTA GGA TTT TTA TAT TTT GAC ACT GAA GAC GTT CCA GGA AAC GCT GGA CTT TTT GAT CAG 207
               L   M   A   L   Q   W   V   H   E   N   I   K   L   F   G   G   N   P   N   N   V   T   L
              CTA ATG GCG TTG CAG TGG GTA CAC GAG AAC ATT AAA TTA TTT GGC GGC AAT CCA AAC AAT GTG ACT CTT 276
               F   G   E   S   A   G   A   V   S   V   S   L   H   L   L   S   P   L   S   R   N   L   F
              TTC GGT GAA TCT GCA GGC GCC GTT TCG GTT TCA CTA CAC TTG CTA TCT CCA TTA AGT AGA AAC CTT TTT 345
               N   Q   A   I   M   E   S   G   S   S   T   A   P   W   A   I   L   S   R   E   E   S   F
              AAC CAA GCA ATC ATG GAA TCA GGA TCC TCA ACA GCA CCT TGG GCA ATT TTG TCA CGG GAA GAA AGT TTT 414
               S   R   G   L   K   L   A   K   A   M   G   C   P   D   D   R   N   E   I   H   K   T   V
              AGT AGA GGA CTT AAA CTA GCA AAG GCA ATG GGA TGT CCA GAT GAC AGA AAC GAA ATA CAT AAA ACA GTC 483
               E   C   L   R   K   V   N   S   S   A   M   V   E   K   E   W   D   H   V   A   I   C   F
              GAG TGC TTA AGA AAG GTG AAC AGT TCA GCA ATG GTT GAA AAA GAA TGG GAC CAT GTG GCT ATA TGT TTC 552
               F   P   F   V   P   V   V   D   G   A   F   L   D   D   H   P   Q   K   S   L   S   T   N
              TTC CCG TTC GTT CCG GTG GTC GAT GGC GCT TTT CTT GAC GAT CAT CCT CAA AAG TCT CTA TCA ACA AAC 621
               N   F   K   K   T   N   I   L   M   G   S   N   S   E   E   G   Y   Y   S   I   F   Y   Y
              AAT TTT AAA AAA ACC AAT ATA CTC ATG GGT AGT AAC TCC GAA GAG GGT TAC TAT TCA ATA TTT TAT TAT 690
                                                                                       F
As_AChE_431F  ... ... ... ... ... ... ... ... ... ... ... ... ... ... ... ... ... ... TTT ... ... ... ...
               L   T   E   L   F   K   K   E   E   N   V   V   V   S   R   E   N   F   I   K   A   I   G
              TTG ACG GAG CTT TTC AAA AAG GAG GAA AAT GTG GTG GTG TCA CGT GAG AAT TTT ATT AAA GCT ATT GGA 759
               Q   L   N   P   N   A   D   A   A   V   K   S   A   I   E   F   E   Y   T   D   W   F   S
              CAA CTT AAT CCA AAT GCA GAT GCG GCG GTT AAA TCG GCT ATA GAG TTT GAA TAC ACG GAT TGG TTT AGC 828
               P   N   D   P   E   K   N   R   N
              CCG AAC GAC CCA GAA AAA AAT CGA AAC                                                         897
```

Figure S1. Diagrammatic representation of S431F and A302S PCR-RFP diagnostic assays.

DNA fragment of the wild type strain As_AChE_wt and the resistant strain As_AChE_431F, amplified using the forward TATAAACGTAGTAGTGCCAAGG and and the reverse GCTTTGGACAAAATGGTCGG primer.

Codons of the resistant strain are shown, only in case of differences.

The first box denotes the site for the A302S mutation and the second box encloses the TCA to TTT mutation (S431F).

Yellow highlight shows Cac8I recognition site for the A302S mutation. Green highlight shows the SspI recognition site for the S431F mutation.
