# Supplementary material for: Survey and Molecular Diagnostics of Target Site Mutations Conferring Resistance to Insecticides in Populations of Aphis spiraecola from Greece
Source: Insects. 2025 Nov 25;16(12):1199. doi: 10.3390/insects16121199 (PMC12733833; doi:10.3390/insects16121199)
Supplement: Supplementary file 1 [file insects-16-01199-s001.zip › Fig_S2.pdf]

```
As_nAChR-wt tattgtaaattcttatttcataggtttttttaatattattctataatttctttatacactgattaaaacatacaaact   80

            gcactaataaagaatggaattattcatataattttatgttttgtttatatcaatttaagttaaataagtttaataatac  160

            gatttgttgaaaaataaatagaatatcaaagtattgattatttataaattattgctaattattaaaatatctaaataaat  240
                                    N  E  K  S  Q  I  M  K  S  N  V  W  L  R  L
            aatatgtgttttacttgttacagAACGAAAAGAGTCAAATAATGAAATCGAACGTTTGGTTGAGACTAgtgagtaccaac  320

            ttaatatatttattttcagtttgtaaatcagaaaattaaaaataaaaagtttcttatcaggcgtattagacgtcttatcc  400

            gactatcatattataaacaactaataccgcttaaaagggaggaaacctacttagcgatttttctct               466
```

Figure S2. Diagrammatic representation of R81T PCR-RFPL diagnostic assay.

DNA fragment of the wild type strain As_nAChR-wt, amplified using the forward TGCATACGTGGTACGTACATAA and the reverse GCTTGACTGCAAACCGTTCA primer. Small letters denote introns and capital letters exons.

The box encloses the site for the R81T mutation.

Yellow highlight denotes BsmAI recognition site for the R81T mutation.
