# Supplementary material for: Survey and Molecular Diagnostics of Target Site Mutations Conferring Resistance to Insecticides in Populations of Aphis spiraecola from Greece
Source: Insects. 2025 Nov 25;16(12):1199. doi: 10.3390/insects16121199 (PMC12733833; doi:10.3390/insects16121199)
Supplement: Supplementary file 1 [file insects-16-01199-s001.zip › Fig_S4.pdf]

```
                                                   E    L    R    Q    Y    N    Q    P    I    I    T    Y    I    P    P    F
As_ACC_wt   AATTTGGTGCATACATTGTTGA TGAA CTA  AGA  CAA  TAC  AAC  CAG  CCA  ATT  ATC  ACA  TAT  ATC  CCA  CCA  TTT
As_ACC_mod  AATTTGGTGCATACATTGTTGA TGAA CTA  AGA  CAA  TAC  AAC  CAG  CCA  ATT  ATC  ACA  TAT  ATC  CCA  CCA  TTT
             G    E    L    R    G    G    A    W    A    V    V    D    T    T    I    N    P    R
As_ACC_wt   GGT  GAA  TTG  CGA  GGA  GGA  GCA  TGG  GCT  GTT  GTA  GAT  ACT  ACT  ATT  AAT  CCA  AGA  CA  TATTGAGATGTA
As_ACC_mod  GGT  GAA  TTG  CGA  GGA  GGA  GCA  TGG  GCT  GTT  GTA  GCT  ACT  ACT  ATT  AAT  CCA  AGA  C

As_ACC_wt   TGCAGATCCAG
```

Figure S4. Diagrammatic representation of A2666V PCR-RFLP PCR-RFLP diagnostic assay.

Primers highlighted in gray amplify an 150 bp gene fragment of A. spiraecola ACCase gene. Reverse primer (highlighted in yellow) creates restriction site for MwoI enzyme (underlined nucleotides) and when combined with forward primer enables the detection of the susceptible A2226 allele. Box denotes the site for the A2666V mutation.
