# Supplementary material for: Survey and Molecular Diagnostics of Target Site Mutations Conferring Resistance to Insecticides in Populations of Aphis spiraecola from Greece
Source: Insects. 2025 Nov 25;16(12):1199. doi: 10.3390/insects16121199 (PMC12733833; doi:10.3390/insects16121199)
Supplement: Supplementary file 1 [file insects-16-01199-s001.zip › Fig_S5.pdf]

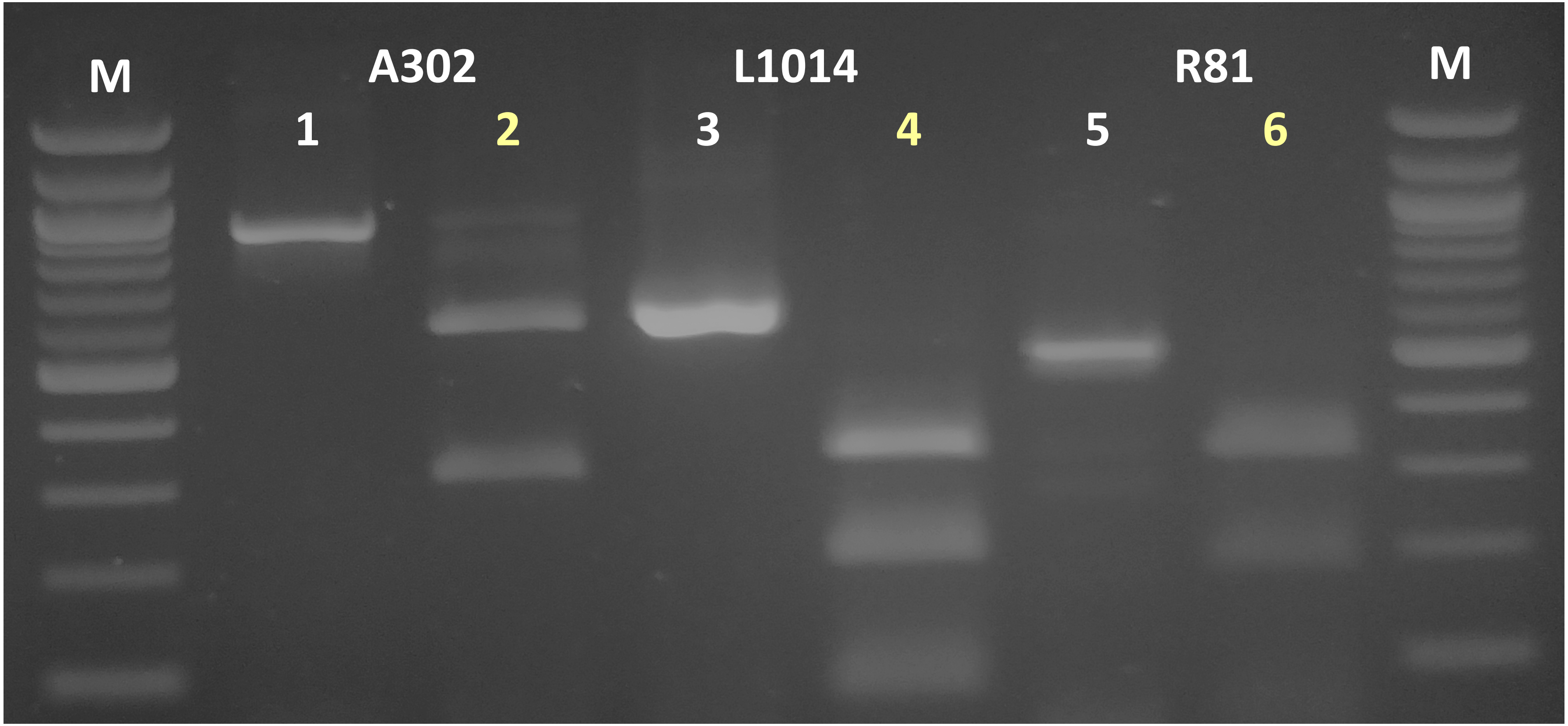

Fig. S5a. PCR-RFLP diagnostic assay for the detection of A302, L1014 and R81 wild type alleles in *Aphis spiraecola*.

Lanes 1, 3, 5: undigested PCR products of AChE, vgsc and nAChR β1 fragments that includes the resistance associated positions A302S, L1014F and R81T respectively. **Lanes 2, 4, 6**: restriction profiles of wild type susceptible allele after enzymatic digestions with Cac8I, BstEII and BsmAI respectively. **M**: Quick-Load 100 bp DNA Ladder, New English Biolabs.

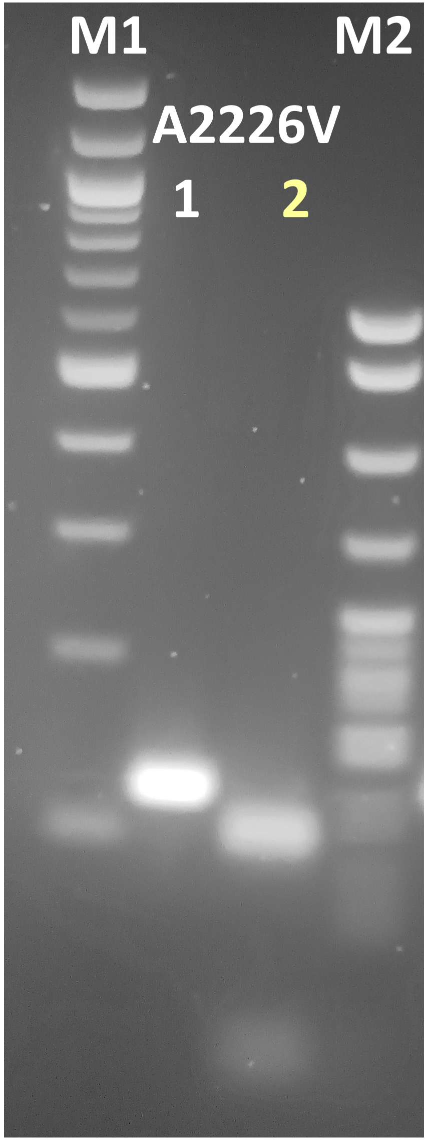

Fig. S5b. PCR-RFLP diagnostic assay for the detection of A2226 wild type allele in *Aphis spiraecola*.

Lane 1: undigested PCR products of ACCase including the resistance associated positions A2226V spirotetramat. **Lane 2**: restriction profiles of wild type susceptible allele after enzymatic digestions with MwoI. **M1**: Quick-Load 100 bp DNA Ladder, New English Biolabs. **M2**: pBR322 DNA-MspI Digest, New English Biolabs.
