# Supplementary material for: Survey and Molecular Diagnostics of Target Site Mutations Conferring Resistance to Insecticides in Populations of Aphis spiraecola from Greece
Source: Insects. 2025 Nov 25;16(12):1199. doi: 10.3390/insects16121199 (PMC12733833; doi:10.3390/insects16121199)
Supplement: Supplementary file 1 [file Fig_S3.pdf › Fig_S3.pdf]

```
                V   L   R   S   F   R   L
As_vgsc_wt CGTA TTG CGT TCA TTT CGT TTG gtgagtatttattttggtgcctaacggtcactatgtgattaaataaaatcacacttca  80
                                         L   R   V   F   K   L   A   K   S   W   P   T   L   N   L
As_vgsc_wt cttatgatttgaaatgtttaactatag CTT CGA GTA TTT AAG TTG GCA AAA TCT TGG CCC ACA CTT AAT CTT 151
            L   I   S   I   M   G   R   T   I   G   A   L   G   N   L   T   F   V   L   C   I   I
As_vgsc_wt TTA ATA TCA ATA ATG GGT CGA ACC ATT GGT GCT TTG GGT AAC CTA ACG TTT GTG TTG TGC ATA ATC 217
            I   F   I   F   A   V   M   G   M   Q   L   F   G   K   N   Y   T
As_vgsc_wt ATA TTT ATA TTT GCY GTT ATG GGT ATG CAG TTA TTT GGA AAA AAC TAC ACAG gtaatctataatgttatm 287
                                                            E   K   V   Y   L   F   K   D   H
As_vgsc_wt    aaacaaacaaaatacgatgrattgcaaataaaattgtgtttttcacag AA AAA GTG TAC TTA TTC AAA GAC CAC 362
            E   L   P   R   W   N   F   T   D   F   L   H   S   F   M   I   V   F   R   V   L   C
As_vgsc_wt GAG CTT CCC CGG TGG AAC TTC ACY GAT TTT TTG CAC TCG TTT ATG ATA GTA TTT CGC GTA TTA TGT 428
            G   E   W   I   E   S   M   W   D   C   L   H   V   G   E   P   T   C   I   P   F   F
As_vgsc_wt GGT GAA TGG ATT GAA TCA ATG TGG GAC TGC TTA CAC GTC GGA GAA CCA ACA TGT ATA CCA TTC TTC 494
            L   A   T   V   V   I   G   N   L   V
As_vgsc_wt TTG GCT ACT GTT GTC ATC GGT AAC CTT GTG gtatgtataagtactgacaatgaataaatatgtattacttaggggaa 571

As_vgsc_wt gatatttaataag                                                                          584
```

Figure S3. Diagrammatic representation of L1014F PCR-RFP diagnostic assay.

DNA fragment of the wild type strain As_vgsc_wt, amplified using the forward CTGCGGGTTACCAAGGACTCTC and the reverse ATGCAAACGGCGAGGTGGAT primer. Small letters denote introns and capital letters exons.

The first box denotes the site for the M918T mutation and the second box encloses the L1014F mutation. Yellow highlights show BstEII recognition sites, the second is absent when the mutation is present.
